# A dual expression plasmid with protegrin-1 compatible with both prokaryotic and mammalian systems^✰^^✰✰^

**DOI:** 10.1016/j.mex.2026.103952

**Published:** 2026-05-10

**Authors:** Manabu Murakami, Hiroshi Koda, Agnieszka M. Murakami, Yasutaka Niwa

**Affiliations:** Department of Pharmacology, Hirosaki University Graduate School of Medicine, 5 Zaifucho, Hirosaki, Aomori 036-8562, Japan

**Keywords:** Antimicrobial peptide, Protein expression, Fluorescence, Plasmid, DNA recombination

## Abstract

Antimicrobial peptide (AMP)-based positive selection is a powerful strategy for rapid gene cloning. A notable example is the use of the *Escherichia coli* (*E. coli*) CcdB toxin to eliminate non-recombinant plasmids. Building on this, dual prokaryotic/mammalian expression vectors like pgMAX and pgMAX-II minimize subcloning by allowing direct expression after a single ligation. However, combining selection modules (e.g., CcdB and Microcin B17) may affect recovery, particularly when their mechanisms both target DNA gyrase.

In this study, we evaluated the feasibility of cloning and expressing the porcine AMP protegrin-1 (PG-1) using an AMP-assisted selection framework. PG-1 is an 18-residue, cysteine-rich cathelicidin that adopts a disulfide bond–stabilized β-hairpin structure. It exhibits potent antibacterial activity by permeabilizing anionic lipid bilayers through pore formation. We demonstrate that protegrin-1 functions as an effective selectable marker for plasmid recombination within the pgMAX-II system, achieving high cloning efficiency. This novel framework enables simple, high-efficiency subcloning with dual-expression capability in both *E. coli* and mammalian cells.

## Graphical abstract

**Figure:**
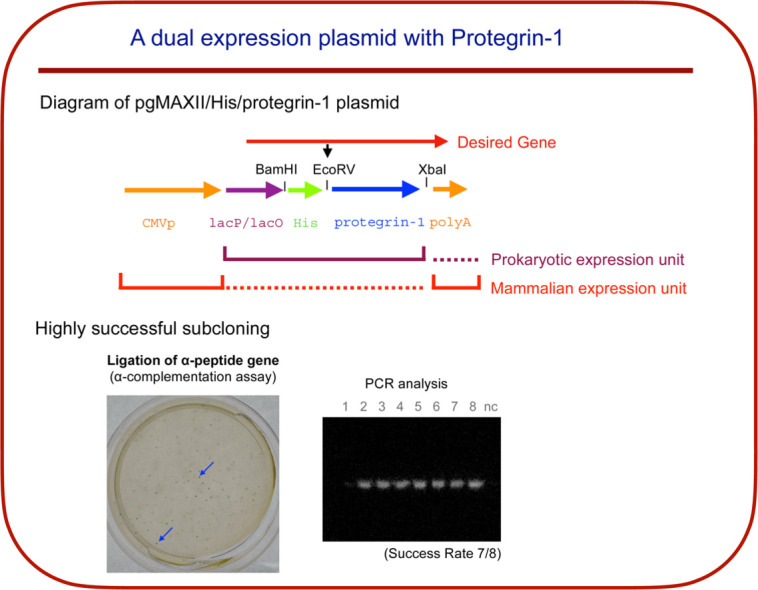

**Key points**

• Protegrin-1 is a cationic, β-hairpin AMP whose membrane-disruptive activity is highly potent.

• Protegrin-1 demonstrates potential for DNA recombination efficiency comparable to CcdB.

• We successfully developed a Protegrin-1-based dual-expression plasmid effective in both prokaryotic and mammalian systems.

Alt-text: Unlabelled box dummy alt text

## Specifications table

**Table:** 

**Subject area**	Pharmacology, Toxicology and Pharmaceutical Science
**More specific subject area**	Gene expression
**Name of your method**	A dual expression plasmid with protegrin-1 compatible with both prokaryotic and mammalian systems
**Name and reference of original method**	pgMAX-II: Murakami, M., et al., A simple, dual direct expression plasmid system in prokaryotic and mammalian cells. PNAS Nexus 2, (2023) 1–3, doi: 10.1093/pnasnexus/pgad139.
**Resource availability**	Not applicable

## Background

Gene cloning and functional evaluation are fundamental in molecular biology, yet workflows often require multiple subcloning rounds between propagation and expression vectors. A common streamlining strategy utilizes the *Escherichia coli* (*E. coli*) CcdB toxin, which targets DNA gyrase by stabilizing cleaved DNA–enzyme complexes [1,2]. In these positive-selection plasmids, target insertion disrupts the toxin, ensuring only recombinant clones survive. However, this approach usually requires subsequent gene transfer into an expression backbone for functional assays.

To bypass these steps, dual-expression plasmids have been developed. Our pgMAX system enables direct expression in *E. coli* after a single ligation and allows conversion into a mammalian vector via blunt-end excision (SwaI/PmeI) and re-ligation [3]. The subsequent pgMAX-II platform advanced this by supporting direct expression in both prokaryotic and mammalian cells [4]. These systems rely on antimicrobial modules like CcdB or Microcin B17 (MccB17), both of which perturb DNA gyrase [5]. Since their mechanisms overlap, combining these toxins within a single framework may affect recombination efficiency and requires evaluation.

In parallel with vector development, antimicrobial peptides (AMPs) provide attractive target genes for functional expression studies. Protegrin-1 (PG-1) is a small, arginine (R) and cysteine (C) -rich porcine cathelicidin (18 amino acids) [6]. PG-1 binds strongly to negatively charged microbial membranes and can permeabilize lipid bilayers; accumulating evidence supports a mechanism involving oligomerization and formation of ion-channel-like pores in anionic membranes, leading to dysregulation of ionic homeostasis and microbial death [7]. Consequently, simple expression of PG-1 in *E. coli* is expected to be toxic and may prevent colony formation.

In this study, we evaluate PG-1 expression within a toxin-assisted framework. We specifically examine how toxin-based selection and the intrinsic antibacterial activity of PG-1 collectively impact recombinant recovery and functional outcomes.

## Method details

### Materials and methods

#### Plasmid construction

**Fig. 1:**
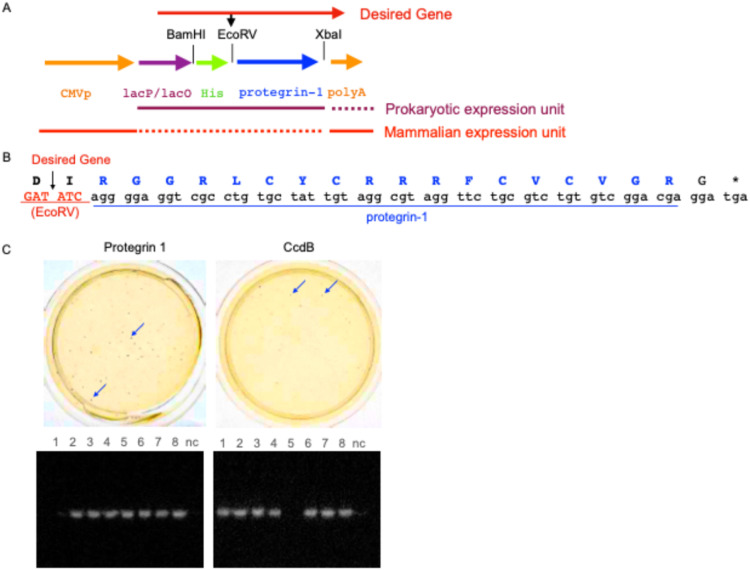
**A. Diagram of the pgMAX-II/His/protegrin-1 dual-expression system.** The pgMAX-II vector features dual promoters: CMV for mammalian expression (orange) and *lac* (Lac P/O) for prokaryotic expression. The protegrin-1 gene (blue arrow) was inserted between *Eco*RV and *Xba*I. Target gene insertion at the *Eco*RV site generates a chimeric fusion protein containing a poly-histidine tag (His; green). **B. Amino acid sequences of protegrin-1**. Amino acid sequences of protein-1 are shown in blue with corresponding DNA sequences for plasmid construction. The *EcoRV* restriction site (GATATC) is highlighted in red. **C. Prokaryotic expression analysis**. Top: α-complementation selection; blue arrows indicate representative blue colonies. Bottom: PCR screening of clones following α-peptide ligation. Seven of eight clones (87.5%) contained the desired insert for both protegrin-1 (left) and CcdB (right). "n" denotes the negative control.

The pgMAX-II plasmid, containing *lac* and CMV promoters, was employed for dual expression in *E. coli* and mammalian cells (Fig. 1A) [3,4]. The protegrin-1 gene (lacking the leader sequence) was amplified using high-fidelity Pfu DNA polymerase (Agilent Technologies, Santa Clara, CA, USA) with primers listed in Supplementary Table 1 [6]. PCR conditions were optimized as follows: 25 cycles of denaturation at 98°C for 10 s, annealing at ∼ 50°C for 30 s, and extension at 72 °C for 30 s. The resulting PCR product was digested with *Eco*RV/*Xba*I, gel-purified, and ligated into pgMAX-II at 16 °C for 30 min.

#### Transformation and purification

Ligated DNA was transformed into Turbo Competent *E. coli* (New England Biolabs, Inc., Ipswich, MA, USA). Competent cells were developed using the K12 strain with the following genotype: [F’ proA+B+ lacIq ∆lacZM15 / fhuA2∆(lac-proAB)glnVgalK16galE15R(zgb-210::Tn10)TetS endA1thi-1∆(hsdS-mcrB)5]. After 1 h recovery in Luria–Bertani (LB) medium (Tryptone 10.0 g/L, Yeast extract 5.0 g/L, NaCl 5.0 g/L, pH 7.0) at 37 °C, cells were plated on LB-ampicillin (150 µg/mL) agar. Single colonies were cultured in 4 mL LB-ampicillin for 12 h. Plasmid DNA was then harvested and purified using an ion-exchange mini kit.

## Method validation

### Subcloning and fluorescent protein expression analysis

#### *α*-complementation assay

To validate recombination and protein expression, a PCR-amplified *lacZ α*-peptide sequence (LQRRDWENPGVTQLNRLAAHPPFASWRNSEE) was inserted into the *Eco*RV site (Fig. 1C). The host *E. coli* strain (K12) carries the *lacZΔM15* mutation, producing the ω-peptide [8]. Following ligation and transformation, clones were grown on LB agar containing ampicillin, X-gal (1.0 mM), and IPTG (1.0 mM) to induce the *lac* operon. After 16 h, blue (*α*-complementation) and white colonies were quantified [4].

The selection mechanism operates as follows: in self-ligated plasmids, IPTG-induced toxin expression inhibits DNA gyrase, preventing colony formation. Only recombinants—where the toxin gene is disrupted by the *α*-peptide sequence (in either orientation)—survive [8]. When the *α*-peptide is inserted in-frame and in the sense direction, a chimeric fusion protein is expressed. This fusion protein exhibits reduced toxicity, allowing *E. coli* proliferation and the formation of blue colonies [8]. Conversely, if the selection module has low inhibitory effects, self-ligation would result in a high frequency of white colonies.

Protegrin-1 (18 amino acids) demonstrated high cloning efficacy, with PCR screening confirming inserts in seven of eight colonies (Fig. 1C, left). The *α*-complementation assay yielded 27.9% blue colonies, indicating potent selection. This performance was comparable to the CcdB control (29.2% blue colonies; Fig. 1C, right).

### Fluorescent protein expression

**Fig. 2:**
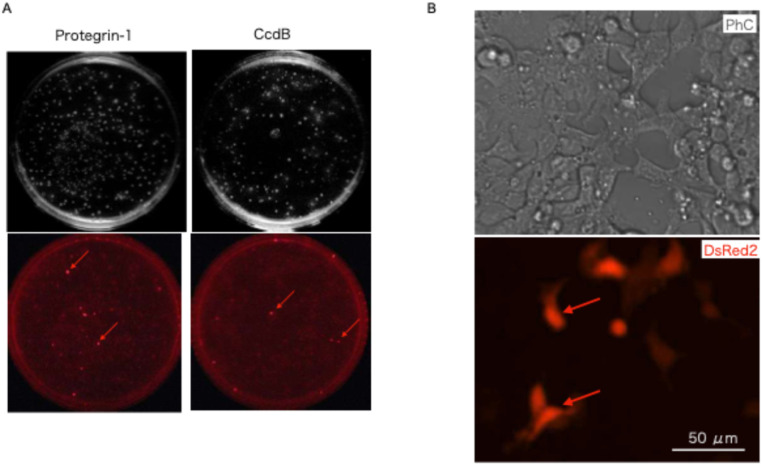
**A. Expression of DsRed2 in *E. coli***. Fluorescent protein expression in the pgMAX-II/His/protegrin-1 system was visualized under green light (excitation: 563 nm) using a red filter (emission: 582 nm). Red arrows indicate colonies successfully expressing DsRed2. **B. Plasmid expression in HEK293 cells**. Representative phase-contrast (PhC; top) and DsRed2 fluorescence (bottom) images are shown. Bright red fluorescence (red arrows) confirms successful transfection and expression of the pgMAX-II/DsRed2/protegrin-1 plasmid in mammalian cells.

A blunt-end DsRed2 fragment (∼700 bp) was amplified using Pfu DNA polymerase with specific primers (DsRed2for: 5′-AAAGCTAGCATGGCCTCCTCCGAGAACGTCATCA-3′; DsRed2rev: 5′-AAAGAATTCAGATCTCAGGAACAGGTGGTG-3′). The product was inserted into the *Eco*RV site of pgMAX-II/protegrin-1. Following ligation and transformation, recombinants were plated on LB agar with ampicillin and IPTG to induce the *lac* operon. After 16 h, colonies were visualized under green light (excitation: 563 nm; emission: 582 nm). Although the frequency of sense-oriented DsRed2 inserts was lower (6.2%) than that observed in the *α*-complementation assay (27.9%), colonies with the correct orientation exhibited distinct red fluorescence (Fig. 2A, red arrows).

The pgMAX-II plasmid contains a modified sequence near the lac promoter [4]. This specific configuration may result in lower promoter activity compared to specialized single-host expression vectors. Therefore, we evaluated IPTG-dependent changes in red fluorescence using the pgMAX-II/His/DsRed2/protegrin-1 plasmid (S3Fig.). A clear dose-dependent response was observed. While background fluorescence inherent to *E. coli* was observed, these results demonstrate that the pgMAX-II system functions effectively as a tunable expression vector.

### Cell culture and transfection of HEK 293 cells

Cell culture and lipofection followed standard protocols [4]. HEK 293 cells (ATCC CRL1573; ATCC, Manassas, VA, USA) were maintained in Dulbecco’s Modified Eagle’s Medium (DMEM) supplemented with 10% fetal bovine serum (FBS). Transfection was performed using Lipofectamine (Invitrogen, Carlsbad, CA, USA). The pgMAX-II/DsRed2/protegrin-1 plasmid was utilized directly for transient expression without further recombination. Bright red fluorescence was observed in HEK 293 cells 48 h post-transfection, confirming the utility of this novel construct as a single-step shuttle vector for both prokaryotic and mammalian systems (Fig. 2B, red arrows).

In this study, the protegrin-1 gene (54 bp) inserted into pgMAX-II is significantly shorter than the conventional CcdB (309 bp). Since standard oligonucleotides are typically ∼60 bases long, the protegrin-1 sequence can be synthesized or amplified in a single PCR step, facilitating its integration into diverse expression plasmids with minimal genetic manipulation.

Generally, shorter peptides are less prone to nonspecific protein-protein interactions. Therefore, compact antimicrobial peptides that retain potent activity are particularly advantageous. In applications involving chimeric toxins—such as antigen-antibody studies—shorter toxin sequences are expected to minimize unintended steric hindrance or off-target interactions. Previously, we established a toxin-sensitivity assay and examined protein-protein interaction using the toxin activity of CcdB [9]. As protegrin-1 possesses a different antimicrobial activity from CcdB, the application of protegrin-1 may reduce false positive clones in the toxin-sensitivity assay.

The minimal color formation observed with the pgMAX-II/α-peptide/PG-1 fusion plasmid under basal condition (0 mM IPTG), coupled with the dose-dependent increase in blue intensity upon IPTG induction (Supplementary Fig. 4), indicates that the leaky expression of the pgMAX-II/protegrin-1 system is low.

Protegrin-1 (PG-1), an 18-amino acid peptide, typically forms two antiparallel β-sheets stabilized by disulfide bonds, exerting its antimicrobial activity by forming oligomeric pores in bacterial membranes. Although we did not directly observe the folding state or disulfide bond formation of PG-1 within the *E. coli* cytoplasm, our functional data provide key insights. First, the immediate growth arrest observed upon IPTG induction suggests that the expressed PG-1 retains its membrane-disrupting activity, implying that it achieves a functional, toxic conformation. Second, our results demonstrate that the insertion of external genes—forming a chimeric fusion with PG-1—allows for normal *E. coli* growth. This suggests that the chimeric protein does not adopt the same toxic pore-forming structure as the native PG-1. While the exact folding mechanism in the environment of the cytoplasm remains to be fully elucidated, the functional switch between the "toxic" (native) and "non-toxic" (chimeric) states provides a robust mechanism for this screening vector.

In this study, we established a dual (prokaryotic and mammalian) expression plasmid vector with protegrin-1 (pgMAX-II/His/Protegrin-1). Due to the antimicrobial activity of protegrin-1, only clones harboring the insert form colonies, thereby enabling efficient subcloning and subsequent protein expression.

Although various subcloning methods, such as T/A cloning, PIPE (polymerase incomplete primer extension), and SLIC (sequence and ligation independent cloning) have recently became available [[10], [11], [12]], most of these are dependent upon PCR. Our pgMAX-II system, featuring antimicrobial peptides, is not only compatible with these methods but can also be applied to standard DNA fragments.

In conclusion, we successfully validated protegrin-1 as an effective selectable marker for plasmid recombination within the pgMAX-II system. Protegrin-1 achieved cloning efficiencies comparable to CcdB (Fig. 1A; Supplementary Fig. 1B). Furthermore, we demonstrated a dose-dependent increase in red fluorescence in response to IPTG. This novel framework enables simple, high-efficiency subcloning with dual-expression utility in both *E. coli* and mammalian cells.

## Limitations

None.

## Ethics statements

This study was conducted with the approval of the Institutional Review Board of Hirosaki University.

## CRediT authorship contribution statement

**Manabu Murakami:** Conceptualization, Methodology, Data curation, Writing – original draft. **Hiroshi Koda:** Investigation. **Agnieszka M. Murakami:** Investigation, Visualization. **Yasutaka Niwa:** Writing – review & editing, Supervision.

## Declaration of competing interest

The authors declare that they have no known competing financial interests or personal relationships that could have appeared to influence the work reported in this paper.

## Data Availability

Data will be made available on request.

## Appendix Supplementary materials

S1 Table. Oligo-DNAs used in this study. S1 Fig. DNA sequences of the protegrin-1 (A) and CcdB (B) peptides, along with their predicted protein sequences. Amino acid sequences of protegrin-1 are indicated in blue. S2 Fig. (A) Sequence of pgMAX-II/His/protegrin-1. (B) Plasmid map pf pgMAX-II/His/protegrin-1 S3 Fig. Dose-dependent induction of DsRed2 by IPTG. (A) Representative images of *E. coli* clones harboring the pgMAX-II/His/DsRed2/protegrin-1 system. Images were captured under white light (left panel) at the indicated IPTG concentrations. Fluorescent protein expression was visualized under green excitation light using a red emission filter (right panel, Red Fluorescence). (B) Quantitative analysis of red fluorescence intensity in response to IPTG. Fluorescence levels were quantified using ImageJ software (https://imagej.net/). S4 Fig. Dose-dependent induction of blue color by IPTG To evaluate the basal expression level of the pgMAX-II/protegrin-1 plasmid, we constructed a pgMAX-II/α-peptide/PG-1 fusion plasmid, which enables the induction of α-complementation by IPTG. *E. coli* cells harboring the recombinant plasmid were grown under several conditions: negative control (nc; no X-gal or IPTG), basal (with X-gal, without IPTG), and IPTG-containing media. After incubation, 1.0 ml of each culture was harvested by centrifugation (5000 x*g*, 1 min). The intensity of the blue color was then evaluated. As expected, *E. coli* showed no blue color under basal conditions (without IPTG). In contrast, IPTG induced blue color formation in a dose-dependent manner. The inset shows the overall appearance of the inverted Eppendorf tubes. S1 Dataset. Original gel image. Image, application 1

## References

[1] Bernard P. (1995). New ccdB positive-selection cloning vectors with kanamycin or chloramphenicol selectable markers. Gene.

[2] Bernard P., Couturier M. (1992). Cell killing by the F plasmid CcdB protein involves poisoning of DNA-topoisomerase II complexes. J. Mol. Biol..

[3] Murakami M. (2019). A simple and dual expression plasmid system in prokaryotic (E. coli) and mammalian cells. PLoS. One.

[4] Murakami M. (2023). A simple, dual direct expression plasmid system in prokaryotic and mammalian cells. PNAS. Nexus..

[5] Murakami A.M. (2025). A dual expression plasmid with Microcin B17 compatible with both prokaryotic and mammalian systems. MethodsX.

[6] Kokryakov V.N. (1993). Protegrins: leukocyte antimicrobial peptides that combine features of corticostatic defensins and tachyplesins. FEBS Lett..

[7] Capone R. (2010). Antimicrobial protegrin-1 forms ion channels: molecular dynamic simulation, atomic force microscopy, and electrical conductance studies. Biophys. J..

[8] Ullmann A., Jacob F., Monod J. (1967). Characterization by in vitro complementation of a peptide corresponding to an operator-proximal segment of the beta-galactosidase structural gene of Escherichia coli. J. Molec. Bio..

[9] Murakami A.M., Nagatomo K., Miyoshi I., Itagaki S., Niwa Y., Murakami M. (2023). A novel binding site between the voltage-dependent calcium channel CaV1.2 subunit and Cavβ2 subunit discovered using a new analysis method for protein-protein interactions. Sci. Rep..

[10] Holton T.A., Graham M.W. (1991). A simple and efficient method for direct cloning of PCR products using ddT-tailed vectors. Nucleic. Acids. Res..

[11] Klock H.E., Lesley S.A. (2009). The Polymerase incomplete primer extension (PIPE) method applied to high-throughput cloning and site-directed mutagenesis. Methods Mol. Biol..

[12] Li M.Z., Elledge S.J. (2012). SLIC: a method for sequence- and ligation-independent cloning. Methods Mol. Biol..

