# Supplementary material for: A dual expression plasmid with protegrin-1 compatible with both prokaryotic and mammalian systems
Source: MethodsX. 2026 May 10;16:103952. doi: 10.1016/j.mex.2026.103952 (PMC13200040; doi:10.1016/j.mex.2026.103952)

Supplementary Table 1

protegrin1For	sense	AAAGATATCAGGGGAGGTCGCCTGTGCTATTGTAGGCGTAGGTTCTGCG
protegrin1Rev	antisense	AAATCTAGATCCTCGTCCGACACAGACGCAGAACCTACGCCTACAATAG
AlphaFor	sense	CCCTATAGTGAGTCGTATTA
AlphaRev	antisense	AGGCCTCCATTCGCCATTCAGGCTGC
DsRed2for	sense	AAAGCTAGCATGGCCTCCTCCGAGAACGTCATCA
DsRe-d2rev	antisense	AAAGAATTCAGATCTCAGGAACAGGTGGTG

S1 Fig.

A

protegrin-1

R G G R L C Y C R R R F C V C V G R G *
aggggaggtcgcctgtgctattgtaggcgtaggttctgcgtctgtgtcggacgaggatga

B

CcdB

R Q F K V Y T Y K R E S R Y R L F V D V
cggcagtttaaggtttacacctataaaagagagagccgttatcgtctgtttgtggatgta

Q S D I I D T P G R R M V I P L A S A R
cagagtgatattattgacacgccggggcgacggatggtgatccccctggccagtgcacgt

L L S D K V S R E L Y P V V H I G D E S
ctgctgtcagataaagtctcccgtgaactttacccggtggtgcatatcggggatgaaagc

W R M M T T D M A S V P V S V I G E E V
tggcgcatgatgaccaccgatatggccagtgtgccggtctccgttatcggggaagaagtg

A D L S H R E N D I K N A I N L M F W G
gctgatctcagccaccgcgaaaatgacatcaaaaacgccattaacctgatgttctgggga

I *
atataa

S2 Fig.

A

```
gacggatcgggagatctcccgatcccctatggtcgactctcagtacaatctgctctgatgccgcatagttaagccagtatctgctccctgcttgtgtgttggaggtcg
ctgagtagtgcgcgagcaaaatttaagctacaacaaggcaaggcttgaccgacaattgcatgaagaatctgcttagggttaggcgttttgcgctgcttcgcgatgtac
gggccagatatacgcgttgacattgattattgactagttattaatagtaatcaattacggggtcattagttcatagcccatatatggagttccgcgttacataactta
cggtaaatggcccgcctggctgaccgcccaacgacccccgcccattgacgtcaataatgacgtatgttcccatagtaacgccaatagggactttccattgacgtcaat
gggtggactatttacggtaaactgcccacttggcagtacatcaagtgtatcatatgccaagtacgccccctattgacgtcaatgacggtaaatggcccgcctggcatt
atgcccagtacatgaccttatgggactttcctacttggcagtacatctacgtattagtcatcgctattaccatggtgatgcggttttggcagtacatcaatgggcgtg
gatagcggtttgactcacggggatttccaagtctccaccccattgacgtcaatgggagtttgttttggcaccaaaatcaacgggactttccaaaatgtcgtaacaact
ccgccccattgacgcaaatgggcggtaggcgtgtacggtgggaggtctatataagcagagctctctggctaactagagaacccactgcttactggcttatcgaaatta
atacgactcactatagggagacccaagcttGGTACCGAGCTCGGATCGATGACATTTAAATTTTTACGGTTCCTGGGCTTTTGCTGGCCTTTTGCTCACATGTTCTTT
CCTGCGTTATCCCCTGATTCTGTGGATAACCGTATTACCGCCTTTGAGTGAGCTGATACCGCTCGCCGCAGCCGAACGACCGAGCGCAGCGAGTCAGTGAGCGAGGAA
GCGGAAGAGCGCCCAATACGCAAACCGCCTCTCCCCGCGCGTTGGCCGATTCATTAATGCAGCTGGCACGACAGGTTTCCCGACTGGAAAGCGGGCAGTGAGCGCAAC
GCAATTAATGTGAGTTAGCTCACTCATTAGGCACCCCAGGCTTTACACTTTATGCTTCCGGCTCGTATGTTGTGTGGAATTGTGAGCGGATAACAATTTCACAGTTTA
AACCAGGAAACAGCTATGACCATGGGATCCcatcatcatcatcatcatATgaattcagatatcaggggaggtcgcctgtgctattgtaggcgtaggttctgcgtctgt
gtcggacgaggaTCTAGAtaactgatcataatcagccataccacatttgtagaggttttacttgctttaaaaaacctcccacacctccccctgaacctgaaacataaa
atgaatgcaattgttgttgttaacttgtttattgcagcttataatggttacaaataaagcaatagcatcacaaatttcacaaataaagcatttttttcactgcattct
agttgtggtttgtccaaactcatcaatgtatcttaacgcgtcgagtgcattctagttgtggtttgtccaaactcatcaatgtatcttatcatgtctgtataccgtcga
cctctagctagagcttggcgtaatcatggtcatagctgtttcctgtgtgaaattgttatccgctcacaattccacacaacatacgagccggaagcataaagtgtaaag
cctggggtgcctaatgagtgagctaactcacattaattgcgttgcgctcactgcccgctttccagtcgggaaacctgtcgtgccagctgcattaatgaatcggccaac
gcgcggggagaggcggtttgcgtattgggcgctcttccgcttcctcgctcactgactcgctgcgctcggtcgttcggctgcggcgagcggtatcagctcactcaaagg
cggtaatacggttatccacagaatcaggggataacgcaggaaagaacatgtgagcaaaaggccagcaaaaggccaggaaccgtaaaaaggccgcgttgctggcgtttt
tccataggctccgcccccctgacgagcatcacaaaaatcgacgctcaagtcagaggtggcgaaacccgacaggactataaagataccaggcgtttccccctggaagct
ccctcgtgcgctctcctgttccgaccctgccgcttaccggatacctgtccgcctttctcccttcgggaagcgtggcgctttctcaatgctcacgctgtaggtatctca
gttcggtgtaggtcgttcgctccaagctgggctgtgtgcacgaaccccccgttcagcccgaccgctgcgccttatccggtaactatcgtcttgagtccaacccggtaa
gacacgacttatcgccactggcagcagccactggtaacaggattagcagagcgaggtatgtaggcggtgctacagagttcttgaagtggtggcctaactacggctaca
ctagaaggacagtatttggtatctgcgctctgctgaagccagttaccttcggaaaaagagttggtagctcttgatccggcaaacaaaccaccgctggtagcggtggtt
tttttgtttgcaagcagcagattacgcgcagaaaaaaaggatctcaagaagatcctttgatcttttctacggggtctgacgctcagtggaacgaaaactcacgttaag
ggattttggtcatgagattatcaaaaaggatcttcacctagatccttttaaattaaaaatgaagttttaaatcaatctaaagtatatatgagtaaacttggtctgaca
gttaccaatgcttaatcagtgaggcacctatctcagcgatctgtctatttcgttcatccatagttgcctgactccccgtcgtgtagataactacgatacgggagggct
taccatctggccccagtgctgcaatgataccgcgagacccacgctcaccggctccagatttatcagcaataaaccagccagccggaagggccgagcgcagaagtggtc
ctgcaactttatccgcctccatccagtctattaattgttgccgggaagctagagtaagtagttcgccagttaatagtttgcgcaacgttgttgccattgctacaggca
tcgtggtgtcacgctcgtcgtttggtatggcttcattcagctccggttcccaacgatcaaggcgagttacatgatcccccatgttgtgcaaaaaagcggttagctcct
tcggtcctccgatcgttgtcagaagtaagttggccgcagtgttatcactcatggttatggcagcactgcataattctcttactgtcatgccatccgtaagatgctttt
ctgtgactggtgagtactcaaccaagtcattctgagaatagtgtatgcggcgaccgagttgctcttgcccggcgtcaatacgggataataccgcgccacatagcagaa
ctttaaaagtgctcatcattggaaaacgttcttcggggcgaaaactctcaaggatcttaccgctgttgagatccagttcgatgtaacccactcgtgcacccaactgat
cttcagcatcttttactttcaccagcgtttctgggtgagcaaaaacaggaaggcaaaatgccgcaaaaaagggaataagggcgacacggaaatgttgaatactcatac
tcttcctttttcaatattattgaagcatttatcagggttattgtctcatgagcggatacatatttgaatgtatttagaaaataaacaaataggggttccgcgcacat
ttccccgaaaagtgccacctgacgtc
```

B

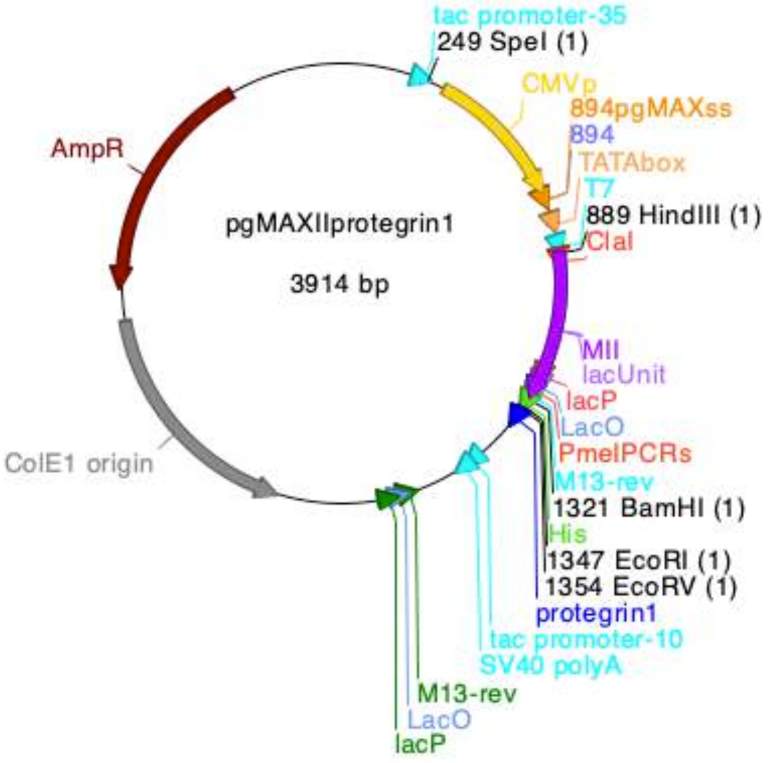

S3 Fig.

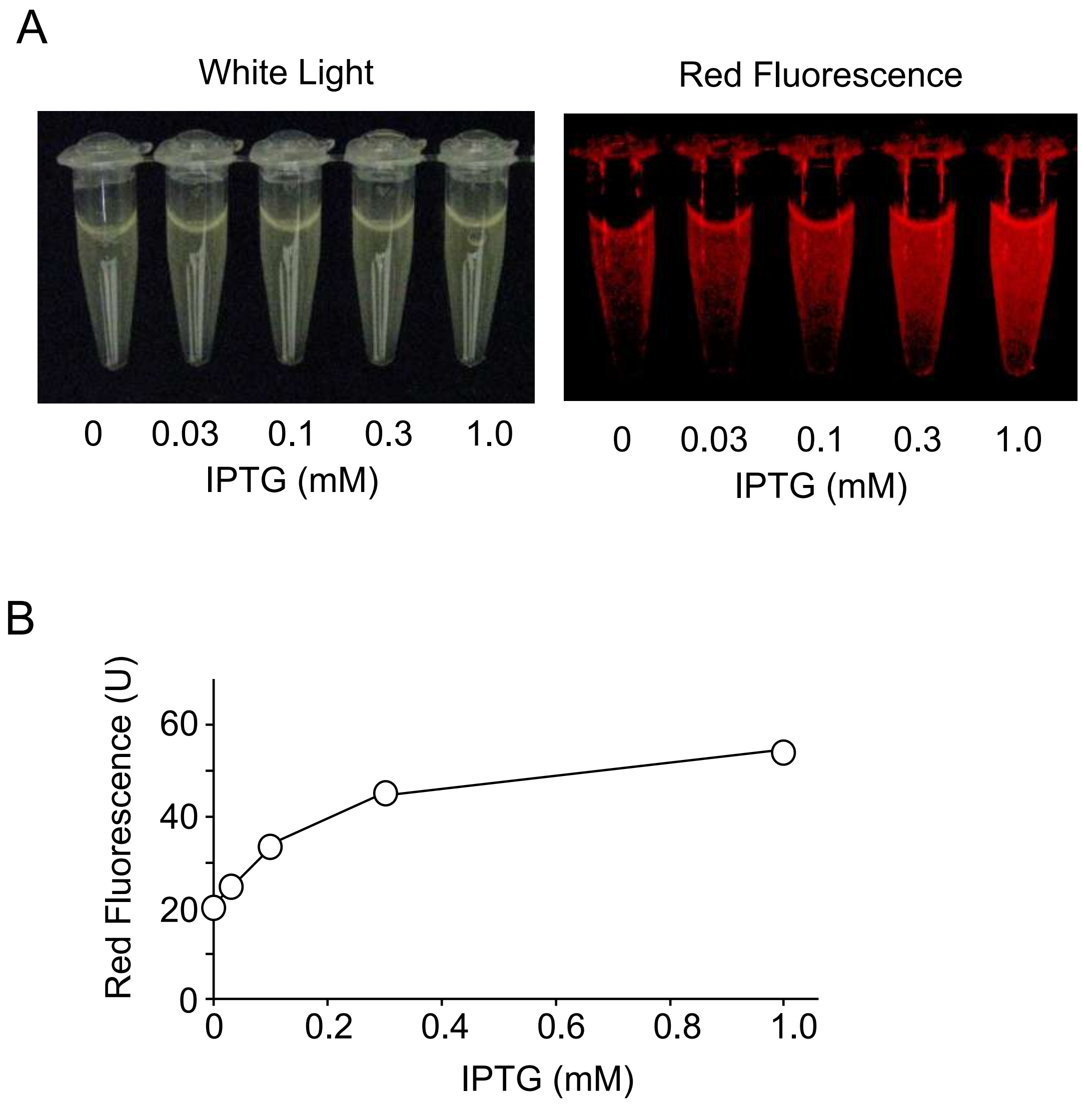
A
White Light
Red Fluorescence
0
0.03
0.1
0.3
1.0
IPTG (mM)
0
0.03
0.1
0.3
1.0
IPTG (mM)
B
Red Fluorescence (U)
0
20
40
60
0
0.2
0.4
0.6
0.8
1.0
IPTG (mM)

S4 Fig.

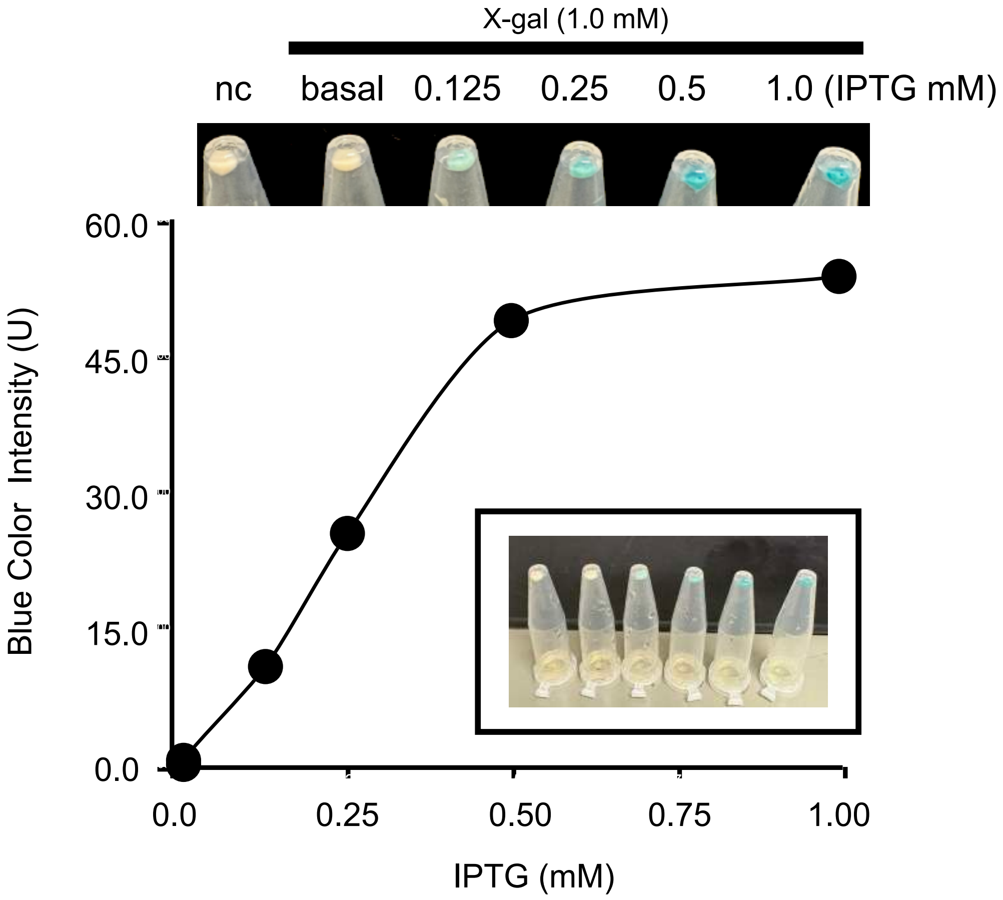
X-gal (1.0 mM)
nc
basal
0.125
0.25
0.5
1.0 (IPTG mM)
Blue Color Intensity (U)
60.0
45.0
30.0
15.0
0.0
0.0
0.25
0.50
0.75
1.00
IPTG (mM)

S1 Dataset

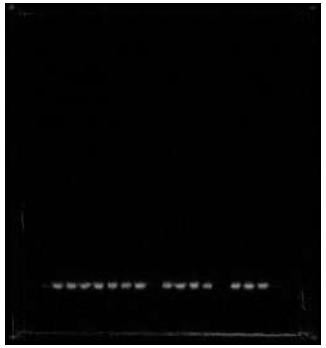

Supplement: Supplementary file 1 — S1 Table. Oligo-DNAs used in this study. S1 Fig. DNA sequences of the protegrin-1 (A) and CcdB (B) peptides, along with their predicted protein sequences. Amino acid sequences of protegrin-1 are indicated in blue. S2 Fig. (A) Sequence of pgMAX-II/His/protegrin-1. (B) Plasmid map pf pgMAX-II/His/protegrin-1 S3 Fig. Dose-dependent induction of DsRed2 by IPTG. (A) Representative images of E. coli clones harboring the pgMAX-II/His/DsRed2/protegrin-1 system. Images were captured under white light (left panel) at the indicated IPTG concentrations. Fluorescent protein expression was visualized under green excitation light using a red emission filter (right panel, Red Fluorescence). (B) Quantitative analysis of red fluorescence intensity in response to IPTG. Fluorescence levels were quantified using ImageJ software (https://imagej.net/). S4 Fig. Dose-dependent induction of blue color by IPTG To evaluate the basal expression level of the pgMAX-II/protegrin-1 plasmid, we constructed a pgMAX-II/α-peptide/PG-1 fusion plasmid, which enables the induction of α-complementation by IPTG. E. coli cells harboring the recombinant plasmid were grown under several conditions: negative control (nc; no X-gal or IPTG), basal (with X-gal, without IPTG), and IPTG-containing media. After incubation, 1.0 ml of each culture was harvested by centrifugation (5000 xg, 1 min). The intensity of the blue color was then evaluated. As expected, E. coli showed no blue color under basal conditions (without IPTG). In contrast, IPTG induced blue color formation in a dose-dependent manner. The inset shows the overall appearance of the inverted Eppendorf tubes. S1 Dataset. Original gel image. [file mmc1.pdf]
